# Golden Rules of Surgery

**Published:** 1906-08

**Authors:** 


GOLDEN RULES OF SURGERY. Aphorisms, Observations and Reflections on Surgery and the Art of Surgery. Being a guide for surgeons and those who would become surgeons. By Augustus Charles Bernays, A.M., M.D., Hdlbg., M. R. C. S. Eng., etc. The C. V. Mosby Medical Book Co., Publishers, St. Louis, Mo.

Much valuable condensed information is contained in this little manual. It gives advice, to young men about to become surgeons, on scientific contributions to the literature of medicine and surgery; on ways and means of building up a practice; about fees, and the cloak of superstition that clings to the medical profession. The golden rules are classified under sub-heads, and as a rule, contain sound advice. Under Gonorrhea, however, we note the following: "Never use an injection, unless it be cocaine, if there is much pain, scalding or inflammation." The prompt relief that follows the injection of certain of the organic silver salts refutes, we think, this statement.

